# Supplementary material for: Effect of Abscisic Acid (ABA) Combined with Two Different Beekeeping Nutritional Strategies to Confront Overwintering: Studies on Honey Bees’ Population Dynamics and Nosemosis
Source: Insects. 2019 Oct 1;10(10):329. doi: 10.3390/insects10100329 (PMC6835648; doi:10.3390/insects10100329)
Supplement: Supplementary file 1 [file insects-10-00329-s001.pdf]

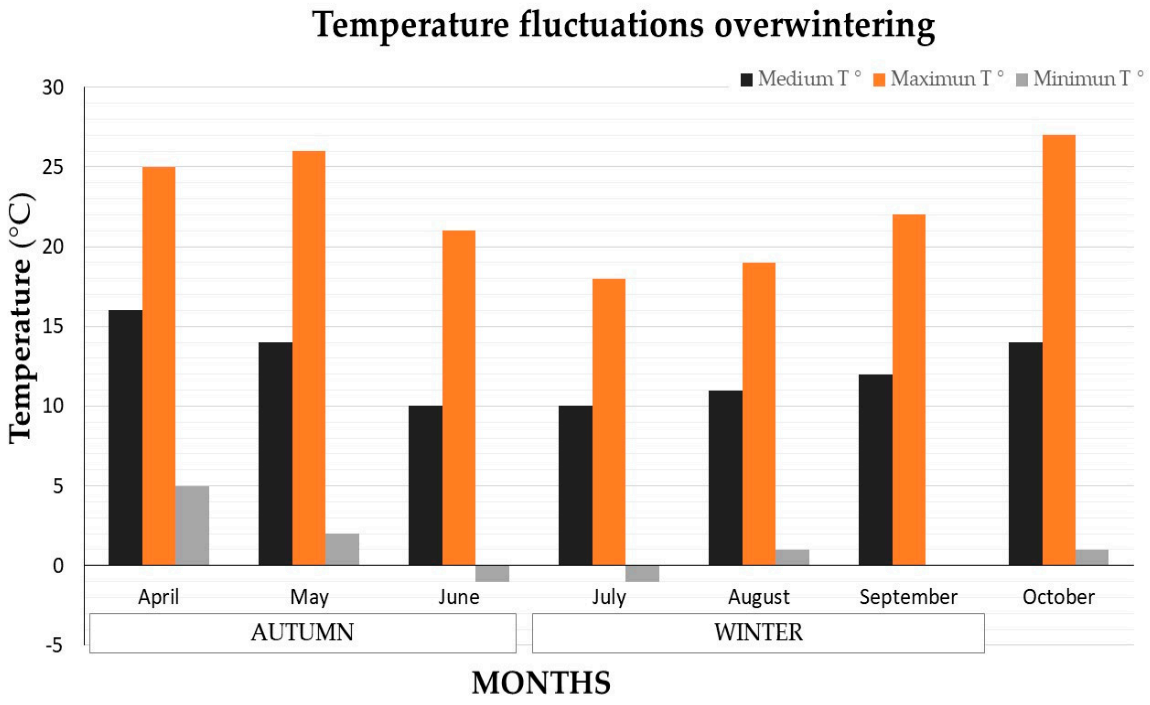

Fig. S1. **Temperature fluctuations during field experiment (autumn-winter, year 2017).** Average temperatures for each month: black bars. Maximum temperature registered each month: brown bars. Minimum temperature registered each month: grey bars.

Oral toxicity to adult honey bees

Methodology

We tested for oral toxicity by adding different concentrations of ABA (FandaChem, CAS Nº 21293-29-8, www.fandachem.com, 90% purity) to the food of worker bees. Twenty-five (25) newly emerged bees were confined in each 15×15×3 cm acrylic box. Each unit was provided with one water feeder and a separate food feeder. Substance doses were incorporated into a powdered sugar and glucose mixture (candy) and supplied *ad libitum* to bees. The concentrations tested were 10 µM, 50 µM and 500 µM of ABA. Nurse bees were maintained under short-term consumption of the different concentration of ABA during 9 successive days. The control was carried out by feeding bees with plain candy (no added ABA.) Each treatment and the control were tested per triplicate (×3). The food was supplied daily and consumption was recorded simultaneously. Using a removable device, the food mass was measured and the amount consumed was divided by the number of live bees.

Statistical analysis

Survival curves (number of live bees versus time for each treatment) were performed by the Kaplan-Meier method (Kaplan and Meier 1958). The non-parametric Log-Rank test was performed to determine differences between survival curves.

Result

The results obtained show that the mortality of bees increases as a function of time for the control group and all tested ABA concentrations. The multiple comparisons by the Log-Rank method between the curves obtained did not show significant differences with respect to the control ($\chi^2 = 1.434$; df = 3; $p = 0.698$).

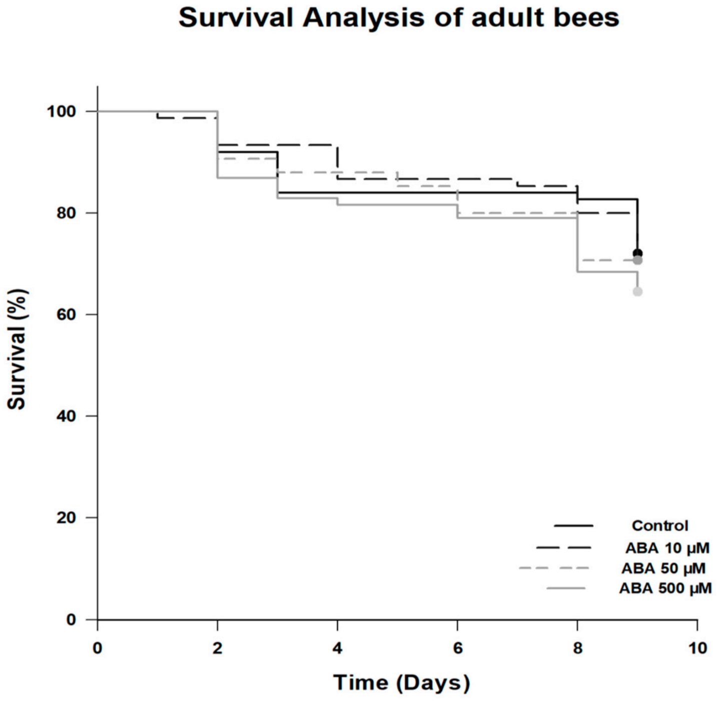

Figure S2. Kaplan-Meier plot for adult honey bee survival. Survival of adult honey bees fed *ad libitum* during 9 days: control (powdered sugar and glucose), ABA 10 µM; ABA 50 µM and ABA 500 µM). In all treatments the curves obtained did not show significant differences with respect to the control (Longrank test, $\chi^2 = 1.434$; df = 3; $p = 0.698$)
